# Probing Gag-Env dynamics at HIV-1 assembly sites using live-cell microscopy

**DOI:** 10.1128/jvi.00649-24

**Published:** 2024-08-13

**Authors:** Frauke Muecksch, Severina Klaus, Vibor Laketa, Barbara Müller, Hans-Georg Kräusslich

**Affiliations:** 1 Department of Infectious Diseases, Virology, Heidelberg University Medical Faculty, Center for Infectious Diseases Research (CIID), Heidelberg, Germany; 2 Chica and Heinz Schaller (CHS) Research Group, Department of Infectious Diseases, Virology, Heidelberg University, Heidelberg, Germany; 3 German Center for Infection Research (DZIF), Partner Site Heidelberg, Heidelberg, Germany; Ulm University Medical Center, Ulm, Germany

**Keywords:** HIV-1, assembly, Env, Gag, PIP2, super resolution imaging, live-cell microscopy

## Abstract

Human immunodeficiency virus (HIV)-1 assembly is initiated by Gag binding to the inner leaflet of the plasma membrane (PM). Gag targeting is mediated by its N-terminally myristoylated matrix (MA) domain and PM phosphatidylinositol 4,5-bisphosphate [PI(4,5)P_2_]. Upon Gag assembly, envelope (Env) glycoproteins are recruited to assembly sites; this process depends on the MA domain of Gag and the Env cytoplasmic tail. To investigate the dynamics of Env recruitment, we applied a chemical dimerizer system to manipulate HIV-1 assembly by reversible PI(4,5)P_2_ depletion in combination with super resolution and live-cell microscopy. This approach enabled us to control and synchronize HIV-1 assembly and track Env recruitment to individual nascent assembly sites in real time. Single virion tracking revealed that Gag and Env are accumulating at HIV-1 assembly sites with similar kinetics. PI(4,5)P_2_ depletion prevented Gag PM targeting and Env cluster formation, confirming Gag dependence of Env recruitment. In cells displaying pre-assembled Gag lattices, PI(4,5)P_2_ depletion resulted in the disintegration of the complete assembly domain, as not only Gag but also Env clusters were rapidly lost from the PM. These results argue for the existence of a Gag-induced and -maintained membrane micro-environment, which attracts Env. Gag cluster dissociation by PI(4,5)P_2_ depletion apparently disrupts this micro-environment, resulting in the loss of Env from the former assembly domain.

**IMPORTANCE:**

Human immunodeficiency virus (HIV)-1 assembles at the plasma membrane of infected cells, resulting in the budding of membrane-enveloped virions. HIV-1 assembly is a complex process initiated by the main structural protein of HIV-1, Gag. Interestingly, HIV-1 incorporates only a few envelope (Env) glycoproteins into budding virions, although large Env accumulations surrounding nascent Gag assemblies are detected at the plasma membrane of HIV-expressing cells. The matrix domain of Gag and the Env cytoplasmatic tail play a role in Env recruitment to HIV-1 assembly sites and its incorporation into nascent virions. However, the regulation of these processes is incompletely understood. By combining a chemical dimerizer system to manipulate HIV-1 assembly with super resolution and live-cell microscopy, our study provides new insights into the interplay between Gag, Env, and host cell membranes during viral assembly and into Env incorporation into HIV-1 virions.

## INTRODUCTION

Human immunodeficiency virus type 1 (HIV-1) assembles and buds at the plasma membrane (PM) of infected cells. Virus morphogenesis is driven by the main structural polyprotein Gag, which binds to the inner leaflet of the PM through its N-terminal matrix (MA) domain and oligomerizes to create the viral assembly lattice. PM targeting of Gag requires MA interacting with PM phosphatidylinositol 4,5-bisphosphate (PI(4,5)P_2_), (1) which is highly enriched in the membrane of the released virus compared to the producer cell PM (2). Gag also mediates the incorporation of the viral RNA genome and other virion-associated proteins including the viral envelope (Env) glycoproteins. Eventually, Gag recruits the cellular endosomal sorting complex required for transport (ESCRT) machinery that facilitates the release of the immature virus (3).

Env is translated as a 160-kDa transmembrane precursor (gp160) at the endoplasmic reticulum, where it trimerizes and becomes glycosylated. Subsequently, Env is transported to the PM via the secretory pathway (4). During transport, cellular furin-like proteases cleave gp160 into the mature surface glycoprotein gp120 and the transmembrane glycoprotein gp41. In the PM, Env trimers diffuse freely and become rapidly internalized and recycled by clathrin-mediated endocytosis (5). Env incorporation into the nascent virion has been shown to depend on direct or indirect interaction between the MA domain of Gag and the long C-terminal tail (CT) of Env (6–13). Specific mutations in MA as well as deletion of the Env CT abolish specific Env incorporation into the virus (6, 7, 9, 10, 13), but the precise mechanism of Env recruitment is currently not understood.

Using super resolution fluorescence microscopy, it has been shown that Env becomes specifically recruited to HIV-1 assembly sites; recruitment is lost upon the deletion of the Env CT (14–16). Furthermore, MA binding to Env CT correlated with MA trimerization (17), and Env incorporation was shown to depend on MA trimerization (18). Compensatory mutations rescuing MA trimer interface mutants were shown to restore impaired Env incorporation and Env trimerization (19, 20). Nanoscale single-particle tracking of Env at the PM indicated that Env is confined to subviral regions of the nascent assembly site, and EnvCT and the Leu12 residue in MA were critical, whereas the induction of membrane curvature was dispensable for the retention of Env in the assembly lattice (21). Recent structural data provided evidence that substitutions of Leu12 and Leu30 induce a conformational change in myristoylated MA, potentially destabilizing the trimer–trimer interactions of the MA lattice (22). While the described studies thus support a connection between MA trimerization and Env incorporation, it is not yet clear whether a direct MA–CT interaction takes place in the MA lattice at viral assembly sites. In fact, the structure of the immature MA lattice revealed that many of those residues reported to lead to defects in Env incorporation when mutated are located close to the intra- and intertrimer interfaces rather than within the holes of the immature MA lattice thought to accommodate the Env CT (23). These residues are, thus, likely to modulate MA trimerization and/or the myristoyl switch but not directly control Env CT binding.

Unexpectedly, Env appears to accumulate mainly in the vicinity of HIV-1 assembly sites, encircling rather than co-localizing with the central Gag cluster (14, 24). This is in line with a study showing that Env distribution in cell-associated release-deficient virions is biased toward the neck, rather than randomly distributed over the virion (16). These observations are consistent with the low number of ca. 7–15 Env trimers in released HIV-1 particles of lab-adapted strains (25–27) and argue for an indirect mechanism of Env recruitment and/or retention. Gag-mediated alteration of the membrane microenvironment may recruit Env molecules to the assembly site in a CT-dependent manner, while Env incorporation into the ordered immature Gag lattice could be hampered by the long CT (151 amino acids per molecule of the trimer). The former hypothesis is consistent with the observed altered lipid composition of the virion membrane compared to the producer cell PM (2, 28–31).

Using a live-cell imaging-compatible rapid chemical dimerizer system (rCDS) that allows for the reversible depletion of PI(4,5)P_2_ from the PM (32), we have previously presented an approach to manipulate the Gag–PM interaction and thereby control Gag assembly in living cells in real time (24). These experiments confirmed that PM recruitment and assembly of Gag are inhibited by PI(4,5)P_2_ depletion and showed that Gag assembly can be rapidly induced by PI(4,5)P_2_ restoration. Furthermore, pre-formed Gag clusters at the PM were rapidly lost upon PM PI(4,5)P_2_ depletion and could be re-induced by PI(4,5)P_2_ restoration (24). While these and other experiments elucidated the dynamics of Gag assembly (24, 33, 34), the dynamics of Env recruitment to viral assembly sites and the mechanism of Env incorporation into virions are less well understood. Here, we exploit the rCDS system to monitor and quantify the dynamics of Env recruitment to nascent viral assembly sites in real time and analyze the effects of Gag assembly site disruption on the associated Env microdomain.

## RESULTS

### Live-cell detection of HIV-1 Env at HIV-1 assembly sites

Our previous analyses of Env PM distribution in fixed cells relied on indirect immunolabeling for Env detection, using the broadly neutralizing monoclonal antibody 2G12 in combination with fluorescently labeled secondary antibodies (14). To avoid antibody-induced clustering and minimize the influence of the large antibodies on Env mobility in our live-cell experiments, purified antigen-binding fragments (Fabs) of 2G12 (2G12Fab) were now directly coupled to the organic fluorophore Abberior STAR RED, generating the reagent 2G12FabSR.

We first performed confirmatory experiments to validate this tool for the detection of Env at HIV-1 assembly sites in fixed and live cells. HeLa Kyoto cells were co-transfected with pCHIV and its derivative pCHIV^EGFP^ (35), which encode all HIV-1 proteins except for Nef, and in the case of pCHIV^EGFP^, a modified Gag polyprotein with enhanced green fluorescent protein (EGFP) inserted between its MA and capsid domains. We have previously shown that the co-expression of equimolar amounts of Gag and Gag.EGFP results in the formation of particles with uncompromised morphology (36). For super resolution fluorescence microscopy analyses, the EGFP moiety was replaced by a self-labeling CLIP-tag, allowing for intracellular Gag staining with the stimulated emission depletion (STED)-compatible dye Atto 590 BC-CLIP (37).

**Fig 1:**
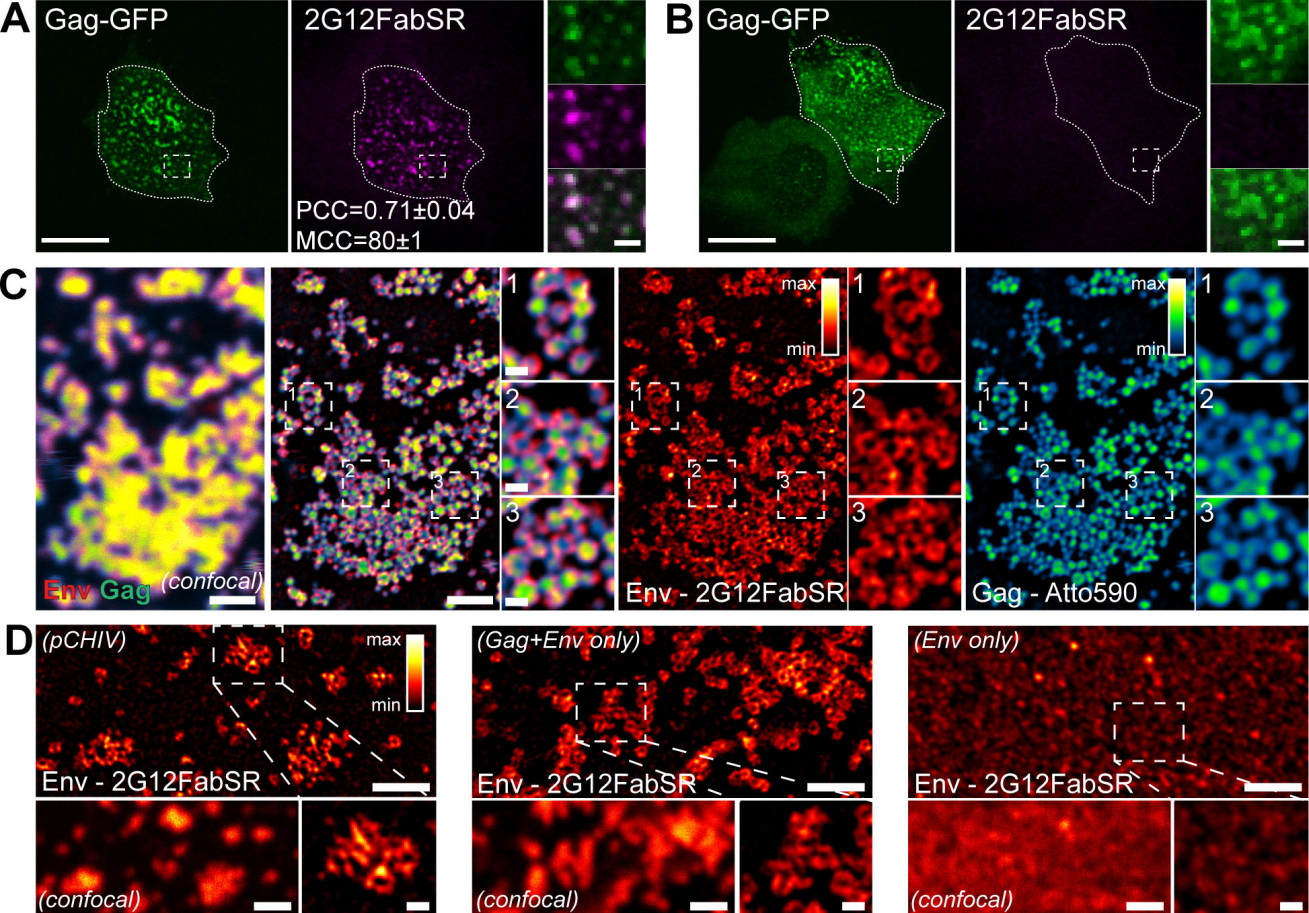
Nanoscopic Env distribution. (A and B) Representative spinning disc confocal microscopy (SDCM) images of the ventral PM of live HeLa cells transfected with pCHIV and pCHIV^EGFP^ (A) or pCHIV(Env-) and pCHIV^EGFP^(Env-) (B). Cells were stained with 2G12FabSR at 22 hpt for 30 min at 37°C and subsequently imaged in the presence of the Fab. Dotted lines indicate outlines of the cells of interest. Scale bars in overviews and enlargements represent 20 and 2 µm, respectively. Green, Gag; magenta, Env. PCC and MCC for Env and Gag ± standard deviation determined for *n* = 3 cells are indicated in panel (A). (C) Micrographs show representative images of the ventral PM of a fixed HeLa Kyoto cell transfected with plasmids pCHIV and pCHIV^CLIP^ acquired in the confocal (left panel) or STED mode. Gag was detected via Atto 590 BC-CLIP (cyan) (30 min labeling in live cells), and following fixation, Env was detected via immunolabeling with 2G12FabSR (red). Legends for multicolor lookup tables (LUTs) are shown in the upper right corner of the single-channel images. Scale bars in overviews and enlargements represent 1 µm and 200 nm, respectively. (D) The micrograph shows a representative super resolution STED image of the ventral PM of fixed HeLa Kyoto cells transfected with plasmids pCHIV and pCHIV^EGFP^ (left panel), pGag and pEnv (middle panel), or pEnv alone (right panel). Env was detected via immunolabeling with 2G12FabSR. A confocal image of the same fields of view is shown for reference. The legend for multicolor LUT is shown in the upper right corner of the left panel. Scale bars in overviews (confocal and STED) and enlargements represent 1 µm and 200 nm, respectively.

In accordance with previous studies (24, 33, 34), transfected cells displayed nascent Gag assemblies at the PM at 22 h post transfection (hpt) (Fig. 1A). The word assemblies in this context refers to the nascent Gag lattice forming a round assembly site in 2D projection images. The labeling of live cells with 2G12FabSR for 30 min prior to image acquisition revealed bright Env clusters co-localizing with round EGFP-labeled Gag assemblies at a diffraction-limited resolution [Pearson’s correlation coefficient (PCC) = 0.71, Manders’ correlation coefficient (MCC) = 80, Fig. 1A], whereby clusters were defined as congregating signals of any shape. Immunolabeling was specific, as cells transfected with a derivative lacking Env [pCHIV(Env-)] and its EGFP-tagged counterpart [pCHIV^EGFP^(Env-)] did not show 2G12FabSR signals, while Gag assemblies were readily detected (Fig. 1B).

To analyze the localization of 2G12FabSR at HIV-1 Gag assembly sites at the nanoscopic scale, we performed STED super resolution analysis of Gag and Env distribution at the PM of HIV-1-expressing HeLa cells. Env and Gag signals again co-localized at the resolution of diffraction-limited confocal microscopy (Fig. 1C). In contrast, STED super resolution microscopy allowed for the analysis of individual assembly sites and revealed Env clusters apparently surrounding rather than co-localizing with the highly condensed Gag signal (Fig. 1C), in accordance with our earlier observations (14, 24). Gag assemblies displayed a diameter of approximately 120–140 nm, as reported for the diameter of HIV-1 particles (38, 39), while apparently ring-shaped Env accumulations surrounding Gag extended beyond this area (Fig. 1C). A direct comparison revealed that Gag is the only viral component required to induce this specific ring-shaped accumulation of Env: Env expressed in the pCHIV-context as well as Env expressed only with Gag, but not Env expressed alone, formed ring-shaped clusters at the PM (Fig. 1D). Instead, Env expressed alone formed smaller, more evenly distributed small punctae at the PM, and no ring-shaped Env accumulations were detectable (Fig. 1D, right panel). In summary, results obtained by Env staining with 2G12FabSR closely resembled those obtained previously by indirect immunostaining (14, 24).

### Live-cell visualization of Env recruitment to nascent assembly sites

We next proceeded to live-cell imaging experiments in HeLa cells expressing pCHIV/pCHIV^EGFP^ at 22 hpt. Incubation with 2G12FabSR 30 min prior to imaging again revealed Env clusters co-localizing with Gag at the PM of HIV-1 expressing cells (Fig. S1A and B). In the continuous presence of 2G12FabSR, the Env PM signal remained high over a period of at least 100 min (Fig. S1A). In contrast, the removal of 2G12FabSR prior to imaging resulted in a substantial decrease of the Env(2G12FabSR) signal over time (Fig. S1B), consistent with continuous endocytotic recycling of Env molecules in HIV-expressing cells (5). Therefore, all further live-cell imaging experiments were performed in the continuous presence of 2G12FabSR. All compounds added during live-cell imaging were diluted in the imaging medium containing 2G12FabSR, keeping the Fab concentration constant throughout image acquisition.

We have previously made use of reversible PI(4,5)P_2_ manipulation at the PM with the rCDS approach to induce Gag assembly (24). In this system, PI(4,5)P_2_ can be depleted from the PM by the addition of a rapid chemical dimerizer (rCD1), which recruits a phosphatase to a CFP-tagged PM anchor; PI(4,5)P_2_ can subsequently be restored by the addition of FK506, which outcompetes rCD1 (32). PI(4,5)P_2_ depletion prior to the onset of Gag assembly (at 4 hpt) inhibited Gag assembly, resulting in Gag accumulation in the cytosol. Viral assembly could then be induced by FK506-mediated restoration of endogenous PM PI(4,5)P_2_ levels, and assembly site formation by targeted induction occurred with similar kinetics as native (non-induced) assembly (24). For the following experiments, we employed a HeLa-derived cell line HeLa_rCDS_ (24), which stably expresses pLCK-ECFP-SNAPf and pmRFP-FKBP-5Ptase (40, 41), the critical components of the rCDS, and allows for the manipulation of HIV-1 Gag assembly by the reversible depletion of PM PI(4,5)P_2_.

**Fig 2:**
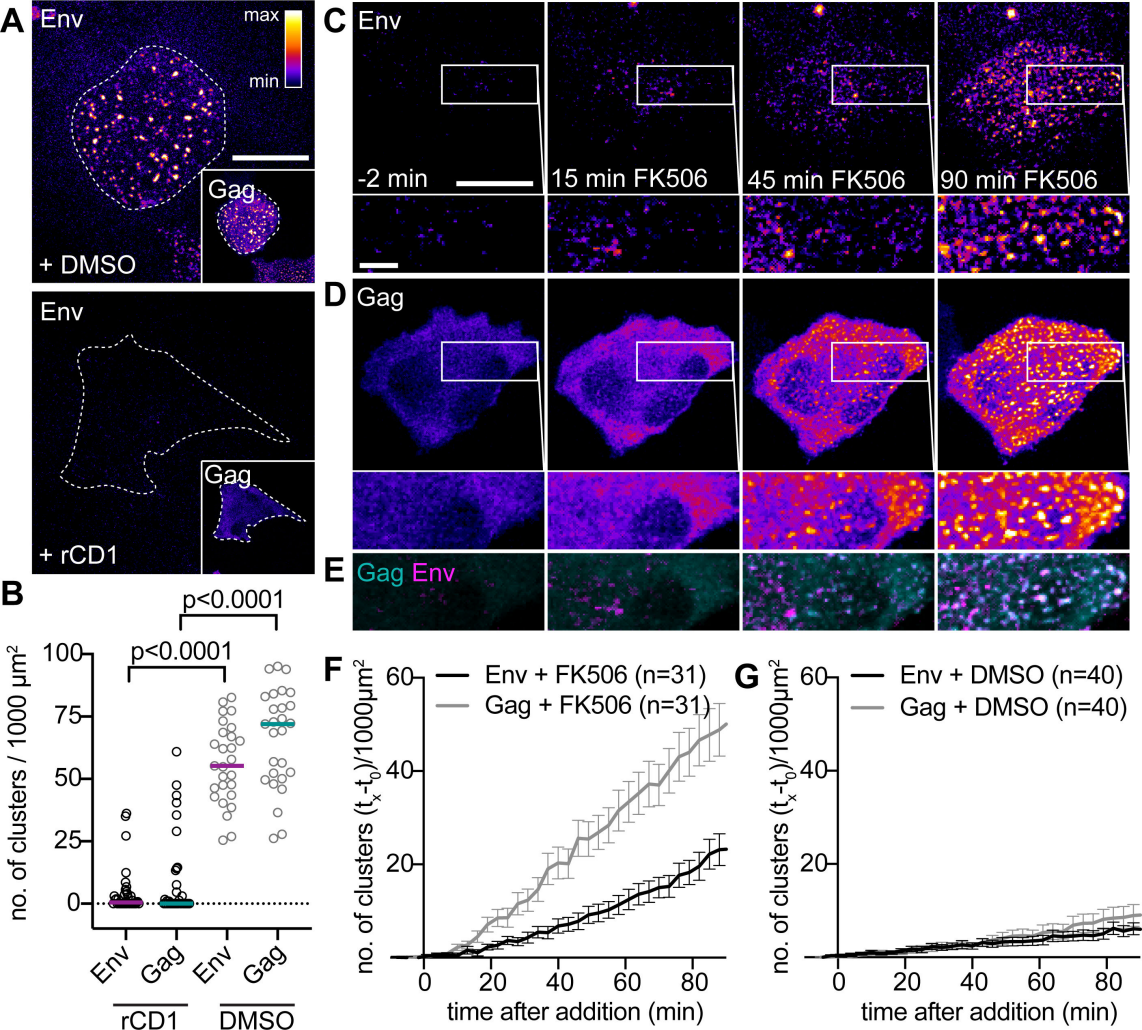
Env recruitment to rCDS-induced HIV-1 assembly sites. (A–B) Effect of PI(4,5)P_2_ depletion on Env and Gag assembly. (A) Representative SDCM images of the ventral PM of HeLa_rCDS_ cells transfected with pCHIV and pCHIV^EGFP^. Cells were treated with 1% DMSO (upper panel) or 1 µM rCD1 (lower panel) at 5 hpt. Prior to imaging at 22 hpt, cells were stained with 2G12FabSR for 1 h. Outlines of the shown cells are indicated with dotted lines. (B) Quantitative analysis of the mean number of Env and Gag clusters per 1,000 µm^2^ for cells treated as in (A). Horizontal lines indicate the median no. of clusters/1,000 µm^2^ for *n* = 37 and *n* = 27 rCD1-treated and DMSO-treated cells, respectively. Statistical significance was evaluated using the Mann–Whitney *U* test (*P* < 0.0001 for Env and Gag, respectively). (C–E) Representative time-lapse SDCM images of the central volume of HeLa_rCDS_ cells transfected with pCHIV and pCHIV^EGFP^. Maximum intensity projections of four focal planes acquired with an axial spacing of 0.5 μm are shown. Cells were treated with 1 µM rCD1 at 5 hpt. Prior to imaging, cells were stained with 2G12FabSR for 1 h. At 22 hpt, cells were treated with 1 μM FK506 for up to 90 min. (E) depicts an overlay of the enlargements shown in (C and D). Single-channel images are displayed in the “fire” LUT, which is shown in the upper right corner of panel (A). Cyan, Gag; magenta, Env in overlays. Scale bars in overviews and enlargements represent 20 and 5 µm, respectively. (F, G) Quantitative analysis of the mean relative number of Gag.EGFP (gray) and Env(2G12FabSR) (black) clusters following FK506 (F) or DMSO (G) addition. Please refer to Materials and Methods for details. Please refer to Fig. S2 for representative images of DMSO-treated control cells. Error bars represent SEM for *n* = 31 FK506-treated cells and *n* = 40 DMSO-treated cells from three independent experiments, respectively.

Since Env does not form PM clusters in the absence of Gag (Fig. 1D), and Gag assembly is dependent on PM PI(4,5)P_2_, we expected that PI(4,5)P_2_ depletion prior to Gag assembly would prevent the formation of Env clusters at the PM as well. As shown in Fig. 2A, this was indeed the case. Automated quantitative analysis of clustered Gag and Env signals (for details, refer to Materials and Methods) yielded significantly fewer Gag/Env clusters in rCD1-treated cells than in DMSO-treated cells (*P* < 0.0001, Mann–Whitney test, Fig. 2B) with less than 10 clusters per 1,000 µm^2^ under PI(4,5)P_2_ depletion conditions for both, Gag and Env. In contrast, DMSO-treated cells displayed approximately 60 clusters/1,000 µm^2^ (Fig. 2B). We then induced Gag assembly and tested whether the recruitment of Env to nascent induced Gag assembly sites could be observed. Treatment of assembly-suppressed cells with FK506 led to the accumulation of Gag assembly sites and clustered Env signals at the PM within approximately 10 min (Fig. 2C through F). This was in stark contrast to results obtained for DMSO-treated control cells, which showed greatly reduced cluster formation at the PM (Fig. 2G; Fig. S2).

**Fig 3:**
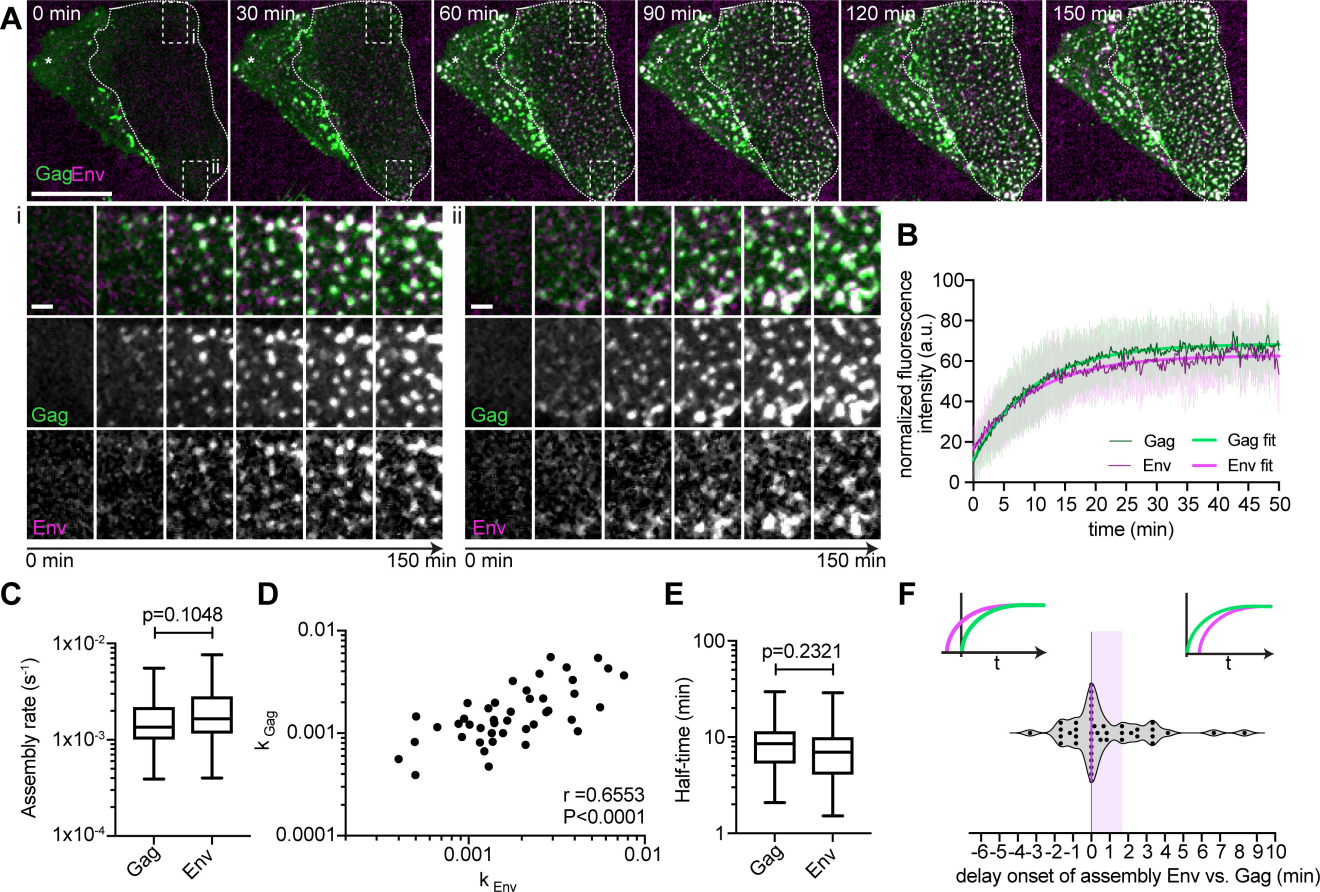
Kinetics of Env recruitment to nascent assembly sites. (A) Representative SDCM images from a high time resolution image series recorded at 10 s/frame. Micrographs show the ventral PM of HeLa_rCDS_ cells transfected with pCHIV and pCHIV^EGFP^. The relevant cell is highlighted with dotted outlines. At 4 hpt, the cells were treated with 1 μM rCD1. Cells were imaged at 22 hpt, and 1 μM FK506 was added at *t* = 0. Green, Gag-EGFP; magenta, Env(2G12FabSR). Enlargements of regions i and ii are shown below. The scale bar in overviews and enlargements represents 20 and 2 µm, respectively. Please refer to Movie S1 for the corresponding complete image sequence. (B) Normalized and averaged Gag and Env HIV-1 assembly traces of 43 individual assembly sites detected at the ventral PM of *n* = 3 cells each. Thin lines represent average Gag (green) and Env (magenta) assembly traces with error bars indicating standard deviation. Single exponential curve fits for Gag and Env are shown in bold green and magenta lines, respectively. (C) Assembly rate constants derived from individual assembly traces by fitting to single exponential equations. Mean assembly rate constants (±SEM) for Gag and Env assembly were 1.86 ± 0.2 × 10^−3^ s^−1^ and 2.23 ± 0.25 × 10^−3^ s^−1^, respectively. Whiskers represent 5–95 percentile (*P* = 0.1048, statistical significance was assessed with the Wilcoxon test). (D) Correlation between Gag and Env assembly rate constants of 43 individual assembly sites. Linear regression fit shown in black (non-parametric Spearman correlation coefficient *r* = 0.6553, *P* < 0.0001). (E) The mean assembly half-times for Gag and Env were 9.2 and 8.5 min, respectively (*P* = 0.2321, statistical significance was assessed with the Wilcoxon test). (F) Delay of Env accumulation onset relative to Gag in 43 assembly sites. The magenta line indicates median Env accumulation delay relative to Gag with the shaded area depicting the 25%–75% interquartile range.

To analyze the dynamics of Env recruitment to HIV-1 assembly sites, we used an SDCM equipped with two identical EMCCD cameras, allowing for the simultaneous acquisition of Gag and Env channels at high spatiotemporal resolution with minimal phototoxicity. Figure 3A shows a representative HeLa_rCDS_ cell subjected to PI(4,5)P_2_ depletion at 4 hpt with pCHIV and pCHIV^EGFP^. Image acquisition was started at 22 hpt, and 2G12FabSR was added 30 min prior to imaging. PI(4,5)P_2_ depletion efficiently inhibited HIV-1 assembly (0 min, Fig. 3A, first panel). FK506 was added at *t* = 0 to rescue PI(4,5)P_2_ PM levels and thereby induce Gag assembly. Images were recorded with a rate of 10 s/frame over a period of 2.5 h. Numerous HIV-1 assembly sites formed during the observation time, with most Gag assemblies appearing co-localized with an Env-specific signal (Fig. 3A; Movie S1). Visual inspection of nascent assembly sites (Fig. 3A, see enlarged regions i and ii), gave the impression that Env started to accumulate simultaneously with or shortly after coinciding Gag assemblies. For quantitative analysis, we performed single virus tracking of nascent assembly sites in the Gag and Env channel.

For this, single assembly sites were tracked, and fluorescence intensity was recorded over time for individual tracked assembly sites. Figure 3B shows normalized and averaged HIV-1 Gag and Env assembly traces derived from assembly sites of three different cells (*n* = 43). No apparent differences between the assembly behavior of Gag and Env were found. For better comparison, Gag and Env intensities from each assembly trace were fitted to single exponential equations to extract assembly rate constants *k*_Gag_ and *k*_Env_ for each individual analyzed assembly site. Mean assembly rate constants derived from averaging all individual *k*-values did not differ significantly between Gag and Env with *k*_Gag_ = 1.86 ± 0.2 × 10^−3^ s^−1^ and *k*_Env_ = 2.23 ± 0.25 × 10^−3^ s^−1^ (*P* = 0.1048, Wilcoxon test, Fig. 3C). Gag and Env assembly rate constants were significantly correlated, with a non-parametric Spearman correlation coefficient of *r* = 0.66 (*P* < 0.0001, Fig. 3D). This observation supports the hypothesis that Env accumulation at Gag assembly sites is a direct consequence of Gag accumulation. Half-times of assembly were not significantly different, with 9.2 min for Gag and 8.5 min for Env (*P* = 0.23, Fig. 3E). Single virus tracking allowed us to assess the starting point of Env accumulation relative to Gag accumulation (Fig. 3F). Importantly, while our analyses cannot detect single Gag or Env molecules congregating at the viral assembly site and detection limits may differ between the different labels, it allowed us to estimate the onset of Env assembly relative to Gag assembly. Gag and Env appeared simultaneously at one-third of all assembly sites analyzed (33%; delay = 0 s). Env was first detected after Gag at 44% of all assembly sites (delay > 0 s), and 23% of all assembly sites showed detectable Env levels prior to Gag detection (delay < 0 s). The latter may be due to sensitivity issues and heterogeneity of labeled Gag incorporation into nascent assemblies, which consist of a mixture of unlabeled and fluorescently labeled Gag. Overall, the median delay of Env detection vs Gag detection was 0 s, with a 25% percentile of 0 s and a 75% percentile of 100 s (Fig. 3F, magenta line indicates the median, with 25%–75% interquartile range highlighted by shaded area). Taken together, the onset and the assembly kinetics of Gag and Env assembly at the host cell PM were largely similar.

### Dissociation of Env clusters upon PI(4,5)P_2_ depletion from the PM

**Fig 4:**
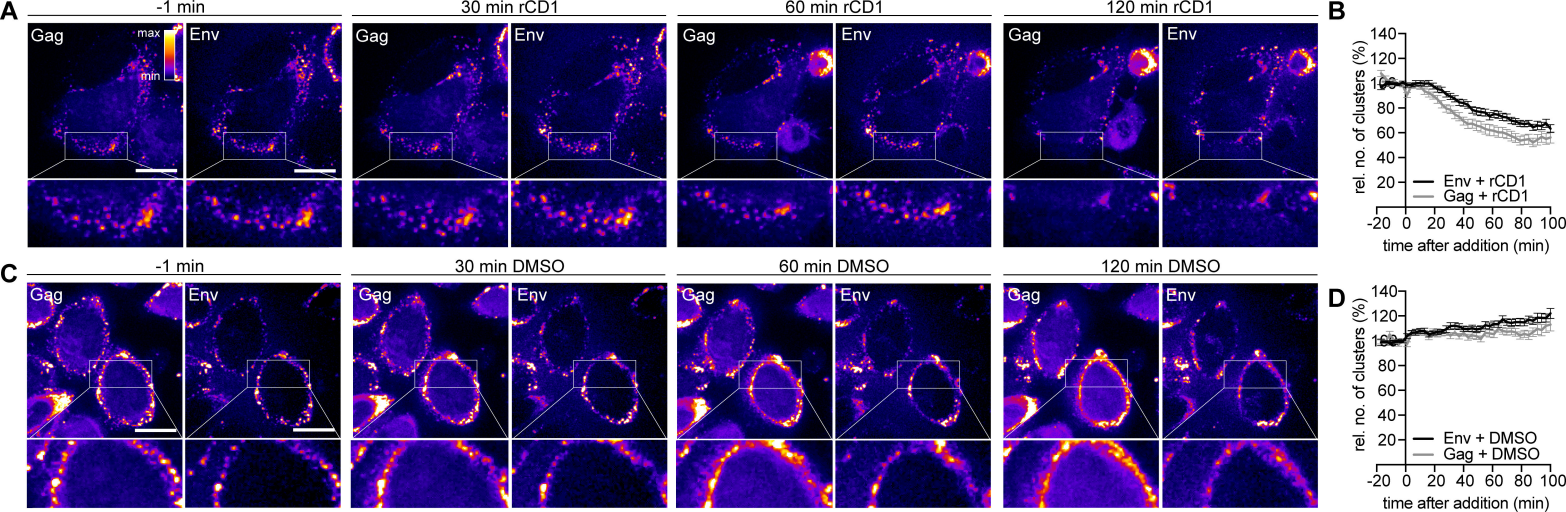
Env clusters dissociate with Gag after PM PI(4,5)P_2_ depletion. (A, C) Representative SDCM images of the central volume of HeLa_rCDS_ cells transfected with pCHIV and pCHIV^EGFP^. Maximum intensity projections of four focal planes acquired with an axial spacing of 0.5 µm are shown. Prior to imaging, cells were labeled with 2G12FabSR for 30 min. At 22 hpt, cells were treated with 1 µM rCD1 (A) or 1% DMSO (C) for up to 120 min in imaging medium containing 2G12FabSR. Scale bars represent 20 µm. Images of Gag and Env (as indicated) are displayed in the “fire” LUT, which is shown in the upper right corner of the left panel in (A). (B, D) Quantitative analysis of the mean relative number of Env-2G12FabSR (black) and Gag.EGFP (gray) clusters following rCD1 (B) or DMSO (D) addition. Error bars represent SEM for *n* = 43 rCD1-treated cells and *n* = 43 DMSO-treated cells from four independent experiments. Please refer to Movies S2 and S3 for the corresponding complete image sequences.

We subsequently aimed to investigate the dynamics of Env clusters at the PM using the targeted removal of pre-formed Gag assemblies through rapid, induced PI(4,5)P_2_ depletion. As previously observed (24), rCD1-induced PI(4,5)P_2_ depletion in cells with pre-existing Gag assembly sites resulted in the rapid dissociation of Gag assemblies from the PM (Fig. 4A, insets). Parallel detection of Env revealed that the associated Env clusters also dissociated after PI(4,5)P_2_ depletion (Fig. 4A and B; Movies S2 and S3). The observed dissociation was specific for rCD1-induced PI(4,5)P_2_ depletion, as the addition of DMSO did not cause any decrease in Gag or Env cluster number (Fig. 4C and D). The quantification of Gag and Env PM cluster numbers over time confirmed the loss of both Gag and Env clusters upon PI(4,5)P_2_ depletion, with the loss of Gag clusters occurring slightly faster (Fig. 4B).

### PI(4,5)P_2_ depletion-mediated Env cluster dissociation is dependent on the MA domain of Gag and Env CT

**Fig 5:**
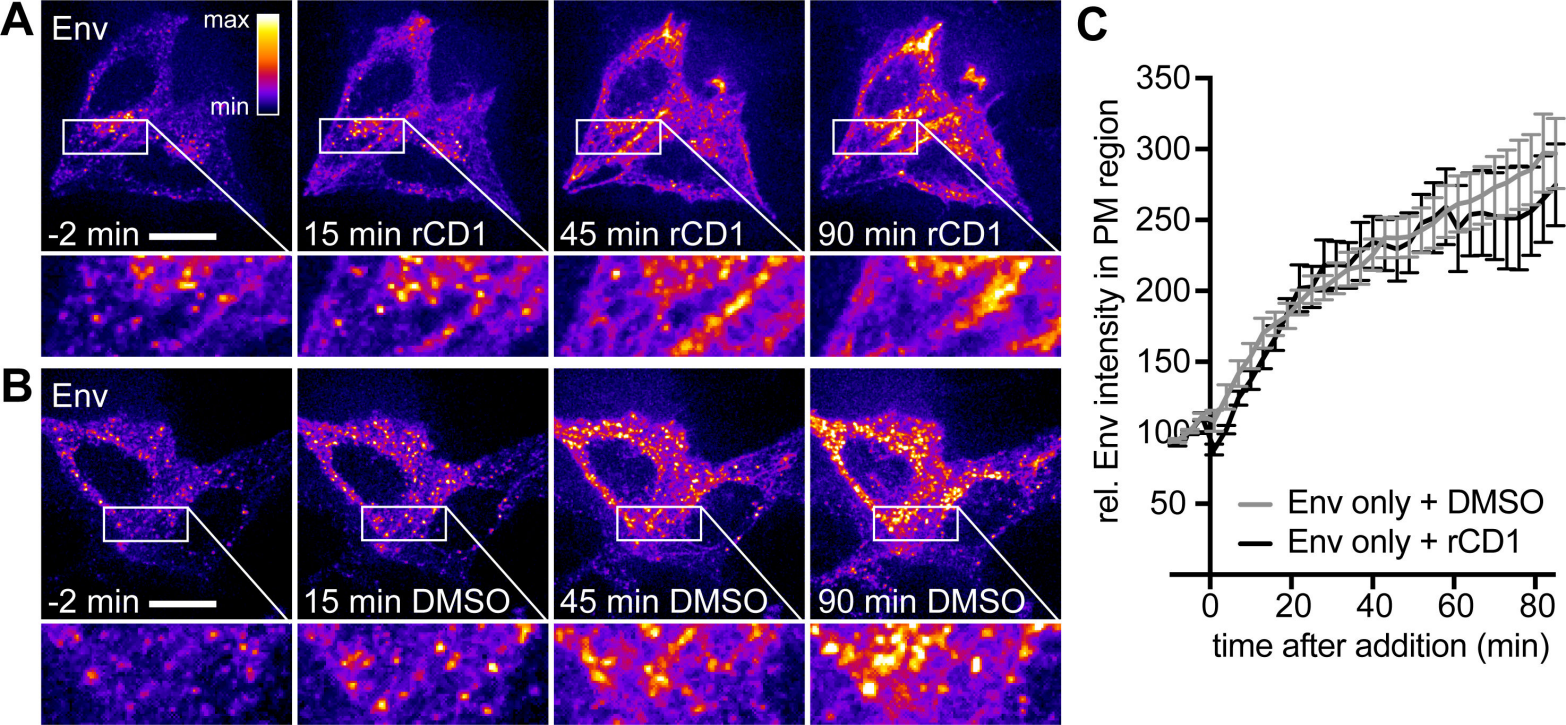
Env alone is not affected by PI(4,5)P_2_ depletion. (A–B) Representative time-lapse SDCM images of the central volume of HeLa_rCDS_ cells transfected with pEnv. Maximum intensity projections of four focal planes acquired with an axial spacing of 0.5 μm are shown. Prior to imaging, cells were stained with 2G12FabSR for 30 min. At 22 hpt, cells were treated with 1 μM rCD1 (A) or 1% DMSO (B) for up to 90 min. Scale bars represent 20 μm. Images of Env are displayed in the “fire” LUT, which is indicated in the upper right corner of the left panel in (A). (C) Quantitative analysis of the relative mean fluorescence intensity of Env expressed alone in the PM region following rCD1 (black) or DMSO (gray) addition. Error bars represent SEM for *n* = 7 rCD1-treated cells from two independent experiments and *n* = 11 DMSO-treated cells from two independent experiments.

To analyze whether the observed loss of Env clusters is directly caused by PI(4,5)P_2_ PM depletion or rather a consequence of PI(4,5)P_2_-mediated dissociation of Gag, we performed PI(4,5)P_2_ depletion experiments in HeLa_rCDS_ cells expressing HIV-1 Env alone. 2G12FabSR staining of HeLa_rCDS_ cells at 22 hpt revealed homogenously distributed Env signals at the PM in addition to prominent intracellular Env punctae (Fig. 5A). Both intracellular clusters and signals at the PM increased in brightness over the observation period of 90 min, independent of whether PI(4,5)P_2_ was depleted or not (compare Fig. 5A and B). For a quantitative assessment of PM-associated Env, we measured the 2G12FabSR channel in the PM area. A comparison of the Env signal at the PM following DMSO or rCD1 addition revealed a very similar extent of signal increase over time, with signals increasing two- to threefold within 80 min of observation (Fig. 5C). These findings show that Env PM association is not directly affected by PI(4,5)P_2_ depletion.

**Fig 6:**
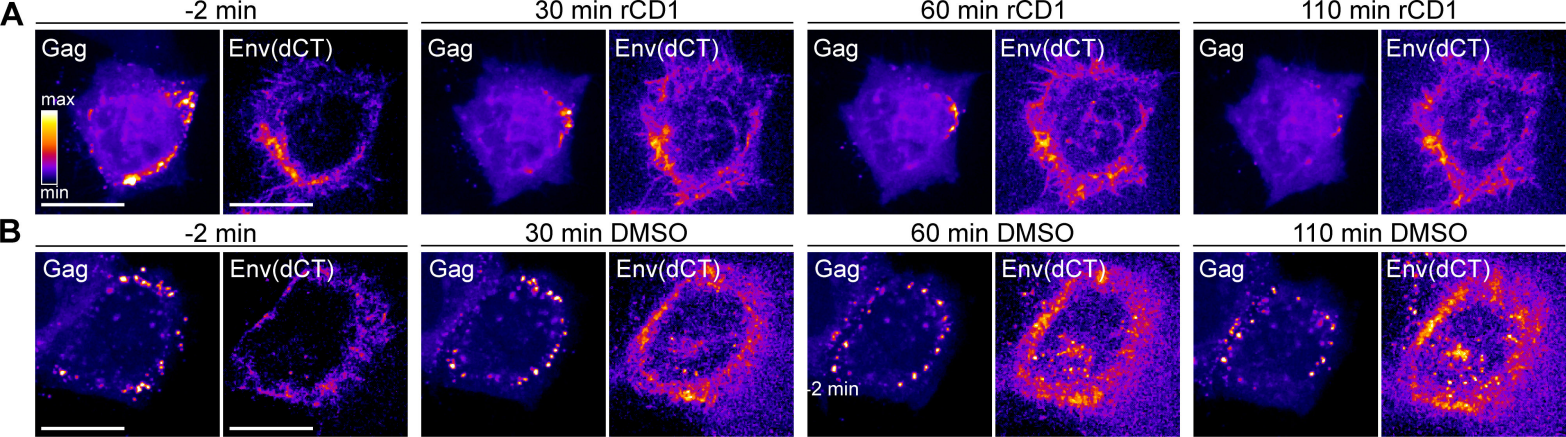
Loss of Env clusters depends on Env CT. (A–B) Representative time-lapse SDCM images of the central volume of HeLa_rCDS_ cells transfected with pCHIV(Env(dCT)) and pCHIV^EGFP^(Env(dCT)). Maximum intensity projections of four focal planes acquired with an axial spacing of 0.5 μm are shown. Prior to imaging, cells were stained with 2G12FabSR for 30 min. At 22 hpt, cells were treated with 1 μM rCD1 (A) or 1% DMSO (B) for 90 min. Images of Gag and Env(dCT) (as indicated) are displayed in the “fire” LUT, which is indicated in the left panel in (A). Scale bars represent 20 μm.

Several lines of evidence have indicated that Env recruitment to HIV-1 assembly sites is CT-dependent (14, 15, 42). We, thus, reasoned that an Env variant with a deletion of the CT (Env(dCT)) should not be affected by PI(4,5)P_2_ depletion, even in the presence of Gag. To test this assumption, HeLa_rCDS_ cells were transfected with the HIV-1-derived constructs pCHIV(Env(dCT)) and pCHIV^EGFP^(Env(dCT)). Env(dCT) displayed a homogenously distributed PM signal with some intracellular punctae (Fig. 6A). As in the previous experiments, rCD1-induced PI(4,5)P_2_ depletion resulted in the loss of Gag assemblies from the PM (Fig. 6A, insets), and this was not observed in DMSO control cells (Fig. 6B, insets). Importantly, there was no detectable decrease of PM-associated Env(dCT) upon PI(4,5)P_2_ depletion in these cells (Fig. 6A). The phenotype of the Env(dCT) variant expressed in the viral context, thus, resembled that of wt Env expressed in the absence of Gag, further supporting the conclusion that the loss of Env upon PI(4,5)P_2_ depletion is mediated by Gag and that Env incorporation in nascent virions is CT-dependent.

We have previously shown that Gag PM localization and late stages of HIV-1 assembly are not PI(4,5)P_2_-dependent in the absence of the MA domain (24), and it is known that mutations in MA affect Env incorporation into nascent virions (7, 9, 10). We, therefore, used the pCHIV(d8SR126) construct, which expresses a Gag variant lacking the globular MA domain while still being myristoylated, and analyzed the effect of PI(4,5)P_2_ depletion on Gag and Env localization. At 22 hpt, Gag and Env expressed in this context showed clusters, which were distributed mainly in the perinuclear region and partially at the PM (Fig. S3). rCD1 addition did not affect Gag or Env localization of the d8SR126 mutant compared to the DMSO control (Fig. S3C and D ).

## DISCUSSION

In striking contrast to other enveloped viruses, HIV-1 incorporates only ca. 7–15 Env trimers into each virion and does not exclude most host cell PM proteins from the viral membrane (25–27). Influenza A, for example, although its spherical particles are similar in size, excludes most host cell PM proteins from its membrane and is densely decorated with 300–400 hemagglutinin and 40–60 neuraminidase trimers (43–45). The sparsity of Env molecules on the HIV-1 surface is likely advantageous for escape from the host immune response (46), but how Env recruitment and incorporation are actually regulated is not yet understood.

The process of Env recruitment and incorporation into the nascent virus is clearly dependent on MA and Env CT, but the observation that the major Env accumulation appears to occur in the periphery of the Gag assembly site would be more consistent with indirect Env recruitment to a specific—Env attracting—membrane microenvironment than with a direct interaction between Env and Gag. Since Env clusters of similar morphology were not observed in the absence of Gag, such a microenvironment would appear to be induced by Gag. Here, we show that when Gag assembly is induced by PI(4,5)P_2_ depletion and restoration, Env is recruited to the periphery of HIV-1 assembly sites concomitant with the formation of the Gag lattice. Our analysis is not sufficiently sensitive to capture the precise timing of the initial accumulation of Gag and Env molecules, but the observed results indicated that the overall dynamics of accumulation at the assembly site were indistinguishable for the two viral proteins. Given this largely simultaneous appearance of Gag and Env at the assembly site, such a lipid microdomain would appear to be formed concomitant with assembly of the Gag lattice.

We next asked whether this putative assembly microdomain remained stable, or was at least transiently maintained, when the Gag lattice was removed from the inner PM leaflet by PI(4,5)P_2_ depletion after HIV-1 assembly sites had already formed. Env rings were rapidly lost from the PM upon Gag removal in an Env CT- and MA-dependent manner. We, thus, conclude that the putative Gag-induced microenvironment requires the presence of MA-anchored Gag and is rapidly lost in its absence. This finding lends additional support to the model that Gag itself shapes and maintains this microenvironment and argues against stable maintenance of the Gag-induced membrane microdomain after the depletion of Gag from the PM. It further suggests that the surrounding Env accumulation also dissipates upon the extracellular release of the nascent virion since the microdomain would no longer be maintained by the Gag lattice.

There is compelling evidence for the enrichment of specific proteins and lipids at the HIV-1 assembly site. The viral envelope contains several proteins, including tetraspanins, at levels that are not correlated with their expression levels on the host cell PM (47–51). Lipidomics analyses further revealed significant differences in lipid composition between the HIV-1 envelope and the host cell PM and suggested a raft-like composition of the HIV-1 membrane, with enrichment of sphingomyelin, cholesterol, and certain phosphoinositides (2, 29–31). Gag sequesters cholesterol at viral assembly sites (52), and a recent microscopy study showed Gag- and curvature-dependent enrichment of sphingomyelin and cholesterol at HIV-1 assembly sites on the PM (53), further supporting viral budding from raft-like membrane microdomains. Proteins have been observed to interact with membrane domains and sort into co-existing fluid phases (54–57). The induction of a liquid-ordered lipid environment by Gag may explain Gag dependence of Env recruitment and maintenance at the viral assembly site despite limited co-localization. A proposed lipid-based partitioning mechanism between liquid-ordered and disordered membrane regions may regulate the selective incorporation of proteins into HIV-1 assembly sites and thereby into released particles (58, 59). The establishment of such microdomains by Gag would facilitate the recruitment of Env toward the assembly site, resulting in Env CT-dependent enrichment, a process that may be enhanced by an interaction between Env CT and cholesterol (60). This membrane domain conducive to Env accumulation must be established concurrently with Gag assembly to achieve the observed simultaneous recruitment of Env to viral assembly sites. Throughout this dynamic process, Env displays ongoing mobility, as evidenced by the need for the continuous presence of anti-Env Fab to sustain Env signals at forming viral assembly sites, highlighting the continuous recycling and recruitment of Env to this membrane microenvironment.

While the Gag-induced membrane microdomain would attract Env, increasing the order of this domain elevates the line tension at the boundary between dissimilar membrane environments. The induction of curvature in the direct vicinity of the Gag assembly domain would decrease the interphase boundary length, thereby reducing the resulting boundary energy and stabilizing the assembly domain (61–64). Such curvature induction can lead to lipid and protein sorting (58, 65). The resulting negative curvature, juxtaposed with the positive curvature of the viral bud itself, forms a distinct geometric configuration at the bud’s neck that affects diffusion, particularly of proteins with large CTs, such as Env. This spatial arrangement would form a barrier at the neck region, limiting Env incorporation into the assembling bud. Such a barrier, potentially combined with the steric exclusion of the bulky Env CT domain from the tight immature Gag lattice, may ultimately hinder Env incorporation into the assembly bud at later stages of assembly. This is in line with findings from Buttler and colleagues, who investigated the subviral angular distribution of Env on cell-associated release-deficient virus using multicolor, 3D super resolution microscopy (16). Their analyses revealed a neck-biased distribution of Env in cell-associated particles, suggesting that Env molecules are trapped at the periphery of assembling Gag lattices, limiting the amount of virion-incorporated Env.

In conclusion, it appears that the Gag-induced membrane microdomain attracts Env molecules to the assembly site, while the increasing negative curvature at the boundary of the growing viral bud, together with the steric restriction imposed by the tight immature Gag lattice, limits the diffusion of Env into the center of the viral bud, collectively resulting in the observed ring-shaped Env clusters at HIV-1 budding sites.

It is an open question why HIV-1 evolved to induce a membrane environment that retains a high number of Env molecules peripheral to the actual assembly site, while only a few Env molecules are incorporated into the virion. HIV-1 can efficiently incorporate heterologous viral glycoproteins that apparently do not require specific recruitment to the viral assembly site (66, 67), a property commonly used to pseudotype HIV-1-based vectors with the vesicular stomatitis virus glycoprotein (68). Furthermore, in some cell types, CT-deficient HIV-1 Env can be incorporated into HIV-1 particles without specific recruitment and has been shown to mediate entry into target cells (13, 69, 70). Therefore, it seems likely that the strong accumulation of wild-type Env in the periphery of the assembly site may be beneficial for the incorporation of Env trimers with their bulky cytoplasmic domains (ca. 50 kDa per trimer) into the tight immature Gag lattice. This is consistent with the increased mobility of Env dCT at the Gag assembly site (15, 16), whereas CD45, a transmembrane protein with a bulky cytoplasmic domain, is selectively excluded from HIV-1 particles (51). Furthermore, studies on the effect of long cytoplasmic C-terminal regions of host cell membrane proteins on HIV-1 incorporation revealed that C-terminal truncation of epidermal growth factor receptor and CD4 increased their respective incorporation into HIV-1 particles (71, 72). Concentrating wild-type Env trimers toward the HIV-1 bud may, thus, facilitate the incorporation of sufficient Env molecules into the virion despite restricted access to the actual assembly site.

Besides facilitating wild-type Env incorporation into the virion, concentrating Env in the PM region of high budding activity may also be important for HIV-1 cell-to-cell transmission. Upon the formation of the virological synapse (73, 74), both Gag and Env localization in the producer cell become polarized toward the target cell, allowing for efficient virus transfer and infection. The formation of the (transiently) stable synapse depends on HIV-1 Env binding to CD4 on the target cell, and this would be less efficient if the majority of Env molecules were incorporated into budding virions and, thus, lost from the PM by virus release. In contrast, the formation of concentrated Env clusters around HIV-1 assembly sites may facilitate synapse formation in the regions of highest budding activity. While these Env clusters would rapidly dissipate upon virus release, they may remain more stable when engaged in the formation of the virological synapse. Further analysis of the Gag-induced lipid sorting and microdomain formation, as well as the mechanism and contribution of peripheral Env accumulation, may, thus, shed light on an important mechanism of HIV-1 transmission *in vivo*.

## MATERIALS AND METHODS

### Chemicals, reagents, plasmids, and cell lines

All chemicals and reagents were purchased from commercial sources unless noted otherwise. rCD1 was synthesized according to previously described procedures (40). FK506 was purchased from LC Laboratories (Woburn, MA, USA).

2G12FabSR was generated from monoclonal anti-gp120 antibody 2G12 (Polymun Scientific Cat#AB002; RRID:AB_2661842) by papain digestion and subsequent protein A purification of the resulting Fab fragment (Pierce Fab Preparation Kit Cat#44985, Thermo Fisher Scientific, Waltham, USA), which was then coupled to Abberior STAR RED NHS (Abberior Instruments GmbH, Göttingen, Germany). Atto 590 BC-CLIP was kindly provided by Janina Hanne.

Plasmid pCHIV, which expresses all HIV-1 NL4-3 proteins except for Nef under the control of a CMV promotor, and its derivatives pCHIV^EGFP^ and pCHIV^CLIP^ were described previously (35, 37). pCHIV MA deletion mutants pCHIVd8-126SR (kindly provided by Martin Obr) and pCHIVd8-126SR^EGFP^ were described before (24). Env(ΔCT) mutants pCHIVEnv(ΔCT) and pEnv(ΔCT) were described previously (14), as well as the Env-deficient pCHIV variants pCHIV(Env-) and pCHIV^EGFP^(Env-) (35).

The cell line HeLa_rCDS_ is derived from HeLa Kyoto cells and stably expresses LCK-ECFP-SNAPf and mRFP-FKBP-5Ptase and was described previously (24).

### Cell culture and transfection

HeLa Kyoto (RRID:CVCL_1922) and HeLa_rCDS_ cells (24) were cultured at 37°C and 5% CO_2_ in Dulbecco’s modified Eagle’s medium (DMEM; Invitrogen) supplemented with 10% fetal calf serum (FCS; Biochrom), 100 U/mL penicillin, and 100 µg/mL streptomycin. The cell line identity of HeLa Kyoto cells has been authenticated using STR profiling (Promega PowerPlex 21 Kit; carried out by Eurofins Genomics, Ebersberg, Germany). Cell lines were grown from mycoplasma-free liquid nitrogen stocks. Passaged cells in culture in the lab are monitored regularly (every 4 months) for mycoplasma contamination using the MycoAlert mycoplasma detection kit (Lonza, Rockland, USA), and cell lines used here were contamination-free.

For microscopy experiments, cells were seeded on slides within eight-well Lab-Tek chambered coverglass systems (Thermo Scientific, Waltham, USA) or Ibidi µ-Slide 8 Well Glass bottom slides (Ibidi, Planegg, Germany). At about 50% confluence, cells were transfected using Turbofect (Thermo Scientific, Waltham, USA) according to the manufacturer’s instructions. 0.5 or 0.75 µg DNA was transfected per Labtek or Ibidi well, respectively. Tagged pCHIV derivatives were transfected in an equimolar ratio with their non-labeled counterpart.

At 4 hpt, the transfection mixture was replaced by fresh medium.

### Preparation of Env-stained live-cell samples

Thirty minutes prior to imaging, Env was labeled with 2G12FabSR in imaging medium (DMEM high glucose w/o phenol red w/o glutamine supplemented with 10% FCS, 4 mM GlutaMAX, 2 mM sodium pyruvate, 20 mM HEPES pH 7.4, 100 U/mL penicillin, and 100 µg/mL streptomycin) at 37°C.

In order to achieve PM PI(4,5)P_2_ depletion prior to Gag accumulation, 1 µM rCD1 was added to the medium at 4 hpt. During image acquisition (performed at 22 hpt), samples were additionally treated with 1 µM FK506 to achieve PM PI(4,5)P_2_ reconstitution. In order to achieve PM PI(4,5)P_2_ depletion after Gag assembly at the PM, transfected samples were treated with 1 µM rCD1 at 22 hpt for a period of 90 min during live-cell imaging.

All compounds were added in imaging medium containing 2G12FabSR. The medium was not exchanged between treatments. One percent DMSO was added instead of rCD1 or FK506 as a vector control, as appropriate.

### CLIP labeling and immunofluorescence staining

For STED imaging, HIV-1 Gag was detected via a CLIP-tag [expressed from pCHIV^CLIP^ (37)]. Live cells were stained with Atto 590 BC-CLIP (kindly provided by Janina Hanne) in imaging medium for 30 min. Cells were washed twice with imaging medium, incubated for an additional 30 min at 37°C, 5% CO_2_ in imaging medium, washed with phosphate-buffered saline (PBS), and fixed for immunostaining.

For immunostaining, cells were fixed at 24 hpt for 15 min with 4% paraformaldehyde in PBS. Samples were washed with PBS and blocked for 30 min with 3% bovine serum albumin (BSA) in PBS. Cells were incubated with the indicated primary antibody in 3% BSA in PBS for 2 h, washed with PBS, and incubated with the respective secondary antibody or Fab in 3% BSA in PBS for 1 h. Antibody/Fab combinations were as follows: Env: monoclonal anti-gp120 antibody 2G12 (Polymun Scientific Cat#AB002 RRID:AB_2661842)/Fab Fragment Goat Anti-Human IgG (H+L) (Jackson ImmunoResearch Labs Cat# 109-007-003 RRID:AB_2337555) coupled to Abberior STAR RED NHS (Abberior Instruments GmbH, Göttingen, Germany). Alternatively, cells were incubated with directly labeled 2G12FabSR in 3% BSA in PBS for 2 h. Finally, cells were washed and kept in PBS.

### Microscopy

The majority of spinning disk confocal (SDC) imaging was performed at a PerkinElmer UltraVIEW VoX SDC microscope (PerkinElmer, Waltham, USA) using a 60× Apo TIRF (NA 1.49) oil immersion objective and Hamamatsu C9100-23B EM-CCD camera. Stacks were acquired with a z-spacing of 500 nm. Live-cell imaging was performed at 37°C, 5% CO_2_, 40% humidity using multiposition imaging with an automated stage and the Perfect Focus System (Nikon, Tokyo, Japan) for automated focusing at each time point with a time resolution of 3 min/frame.

High time resolution SDC live-cell imaging (presented in Fig. 3) was performed at an SDC microscope based on Yokogawa CSU-W1 disc with Borealis illumination optimization and Nikon Ti2 microscope stand equipped with a 100× Apochromat TIRF (NA 1.49) oil immersion objective and two identical Andor iXon Ultra 888 Ultra EMCCD cameras. Live-cell imaging was performed at 37°C, 5% CO_2_, 40% humidity with a time resolution of 10 s/frame.

STED imaging was performed at a λ = 775 nm STED system (Abberior Instruments GmbH, Göttingen, Germany), using a 100× Olympus UPlanSApo (NA 1.4) oil immersion objective with 590 and 640 nm excitation laser lines at room temperature. Nominal STED laser power was set to ~30% of the maximal power of 2,400 mW with 10 µs pixel dwell time and 15 nm pixel size.

### Image representation

Representative still images or single frames of image sequences were chosen. Super resolution STED images were deconvolved with a Lorentzian function (full-width half-maximum = 60 nm) using the software Imspector (Abberior Instruments GmbH, Göttingen, Germany). For all images shown, the camera offset value was subtracted, and the contrast and brightness were adapted for optimal display of the image. To eliminate background noise, a 0.5-px median filter was applied to all SDC images. Images are shown in grayscale or pseudo colors. In the latter case, when using multicolor LUTs, the fire LUT was used for single-channel SDC images while different channels of super resolution STED images are shown with the LUTs red hot (referred to as “red”) and green fire blue (referred to as “cyan”).

### Image analyses

#### Colocalization analysis

Co-localization was quantified by calculating Pearson’s (PCC) and Manders’ (MCC) correlation coefficients. For this, only those optical sections representing the part of the cell immediately adjacent to the glass substrate were considered. First, the camera offset values were measured outside of the cell area, and the value was subtracted from individual channels. Next, we performed a rolling ball (10 px radius) background subtraction to further remove unspecific cellular background and a mean filter (1 px) to smoothen the signals. To calculate the PCC and MCC, we used the “Colocalization Threshold” plugin in Fiji/ImageJ, which determines thresholds for both channels automatically.

#### Particle analysis of SDC microscopy data

Out of all cells imaged, those that showed the expected rCDS enzyme translocation and maintained a healthy phenotype throughout the whole imaging process were selected for further analysis. Cells that showed clear signs of cell damage, e.g., severe shrinking, were excluded from the analysis.

Particle analysis of SDC microscopy data was done in Fiji (RRID:SCR_002285) (75) on single slices of the ventral PM or maximum intensity projections of four slices located in the middle of the cell, as indicated. All parameters for image processing were kept constant when comparing different data sets. First, the camera offset value was subtracted from all images. To remove the image background and prepare the images for automatic thresholding, single images—or image sequences in the case of time-lapse imaging—were converted to 8-bit images, and the background was subtracted using a rolling ball (radius = 2 px). The objects of interest were automatically thresholded using the Niblack local thresholding method (76) with the following parameters: radius = 10, parameter 1 = 0, and parameter 2 = −25. The Niblack local thresholding method was chosen as it reliably resulted in correct thresholding, even in the absence of any clustered signals (e.g., in assembly-inhibited cells) where other thresholding methods tended to create artifacts. Correct thresholding was visually evaluated for each individual image sequence. A 0.5 px median filter was applied to all images. The region of interest was selected manually by drawing the outline of the respective cell. Finally, the size and number of particles in each cell for every still image or every frame of a time-lapse sequence were determined using Fiji’s Analyze Particles function with the following parameters: size = 2-infinity px², circularity = 0–1. Correct particle identification was confirmed, and values were exported and further analyzed in Excel (Microsoft, Redmond, USA) or GraphPad Prism (GraphPad Software, Inc., La Jolla, USA; RRID:SCR_002798).

For still images, the number of particles detected at the ventral PM was divided by the membrane area and plotted as the number of Gag clusters/1,000 µm^2^.

For time-lapse series recorded at the ventral cell membrane of cells treated with rCD1 or DMSO at 4 hpt (before Gag accumulation), the number of detected particles in the frames before the addition of FK506 or DMSO was averaged and subtracted from all data points to exclude Gag clusters already present before the addition of the respective compound. Values were normalized for the membrane area and plotted as the number of Gag clusters (*t*_*x*_ − *t*_0_)/1,000 µm².

For time-lapse experiments imaged in a central section of cells treated with rCD1 or DMSO at 22 hpt (after Gag assembly at the PM), the number of particles in the frames before the addition of rCD1 or DMSO was averaged and set to 100%. All values were normalized accordingly and plotted as the relative number of Gag clusters over time.

#### Single assembly site kinetics

Time courses of native or induced Gag assembly at individual assembly sites at the ventral PM were extracted from single-plane SDCM time-lapse sequences at a time resolution of 10 s/frame.

The camera offset value was subtracted from all image sequences using Fiji (RRID:SCR_002285) (75), and image sequences were imported to Imaris 8 (Bitplane AG, Zurich, Switzerland). Spot detection and tracking were performed using Imaris’ spot detection module. Within this process, the background was subtracted, and the estimated diameter for spot detection was set to 700 nm. The quality parameter for spot detection was in the range of 75–200, depending on the data set. Tracking was performed using the autoregressive motion algorithm, assuming a maximum distance between frames of 700–1,000 nm, allowing for a maximum gap size of four frames and a track duration above 300 s. Filling gaps was disabled. Out of the detected spots, those that increased in mean intensity and reached a plateau phase were selected for further analysis. For averaged assembly curves, the mean intensity values over time were exported to Excel, temporally aligned (the beginning of each track was set to *t* = 0), and normalized (smallest value = 0, highest value = 100) in GraphPad Prism (GraphPad Software, Inc., La Jolla, USA; RRID:SCR_002798). The average normalized fluorescent values (a.u.) including the standard deviation over time were plotted. To calculate assembly rate constants and half-times, single exponential fits to the data (either averaged assembly curves or individual assembly traces) were performed using GraphPad Prism software (GraphPad Software, Inc., La Jolla, USA; RRID:SCR_002798).

#### Quantification of Env PM signals

For a quantitative assessment of PM-associated Env, we measured the 2G12FabSR channel in the PM area. For this, a PM mask was generated based on the PM Anchor CFP signal by image segmentation. The mask was then applied to the 2G12FabSR channel, and the absolute Env signal was measured in the masked area and plotted over time.

### Statistical analysis

Data analysis was performed using GraphPad Prism (GraphPad Software, Inc., La Jolla, USA; RRID:SCR_002798). Values are expressed as the mean ± SEM or mean ± SD, as indicated. The D’Agostino and Pearson test was used to assess normality, and the Wilcoxon test was applied for the statistical analysis of paired data, which did not follow a Gaussian distribution. Values of *P* < 0.05 were considered significant.

## Data Availability

Data presented here, including raw images used for the preparation of composite figures and data tables used for the preparation of graphs, are available from the corresponding author upon request.

## SUPPLEMENTAL MATERIAL

Supplemental material jvi.00649-24-s0001.pdf Figures S1 to S3; legends for Movies S1 to S3.

Movie S1 jvi.00649-24-s0002.mp4 Env recruitment to nascent assembly sites.

Movie S2 jvi.00649-24-s0003.mp4 Env clusters dissociate with Gag after PM PI(4,5)P_2_ depletion.

Movie S3 jvi.00649-24-s0004.mp4 Gag and Env clusters remain unaffected by DMSO treatment.

ASM does not own the copyrights to Supplemental Material that may be linked to, or accessed through, an article. The authors have granted ASM a non-exclusive, world-wide license to publish the Supplemental Material files. Please contact the corresponding author directly for reuse.
