# Supplementary material for: Probing Gag-Env dynamics at HIV-1 assembly sites using live-cell microscopy
Source: J Virol. 2024 Aug 13;98(9):e00649-24. doi: 10.1128/jvi.00649-24 (PMC11406925; doi:10.1128/jvi.00649-24)
Supplement: Supplemental material — Figures S1 to S3; legends for Movies S1 to S3. [file jvi.00649-24-s0001.pdf]

Supplemental Materials for

Probing Gag-Env dynamics at HIV-1 assembly sites using live-cell microscopy

Frauke Muecksch, Severina Klaus, Vibor Laketa, Barbara Müller, Hans-Georg Kräusslich

Correspondence should be addressed to Frauke Muecksch, frauke.muecksch@med.uni-heidelberg.de

This file includes:
Supplemental Figures S1-S3
Supplemental Movie Legends

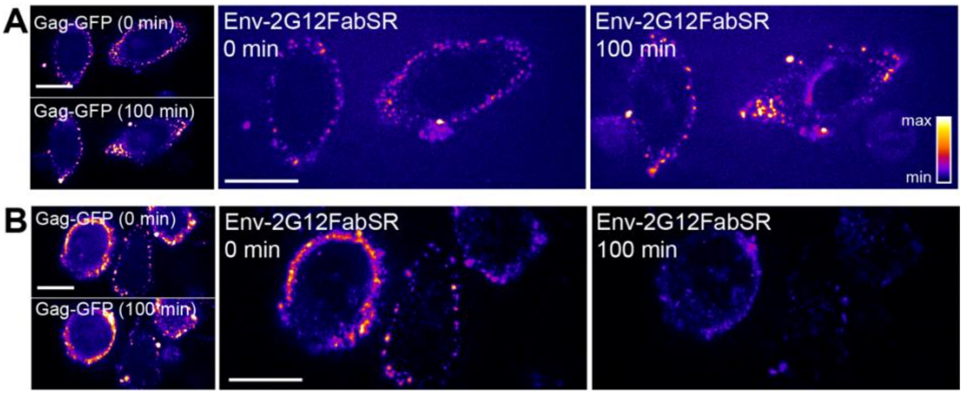

Fig S1 Live-cell labeling of Env. **(A-B)** Representative time-lapse spinning disc confocal microscopy (SDCM) images of the central volume of $HeLa_{rCDS}$ cells transfected with pCHIV and $pCHIV^{EGFP}$. Maximum intensity projections of four central focal planes acquired with an axial spacing of 0.5 µm are shown. At 22 hpt, cells were stained with 2G12FabSR diluted in imaging medium for 30 min. Subsequently (t=0), incubation was either continued in the presence of Fab **(A),** or the medium was replaced by fresh imaging medium lacking 2G12FabSR **(B)**. Images of Gag and Env (as indicated) are displayed in the "Fire" LUT, which is shown in the lower right corner in the right panel in (A).

14

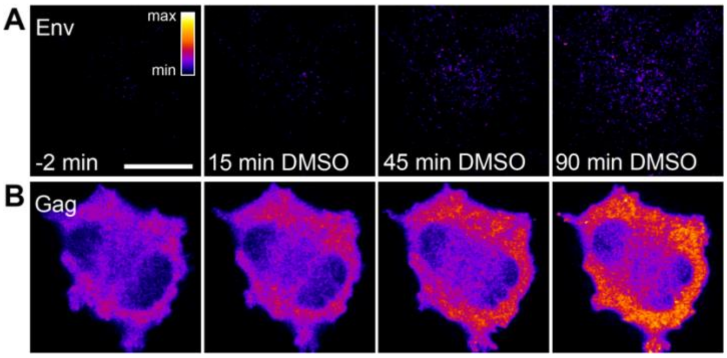

Fig S2 No induction of HIV-1 assembly in rCD1 assembly-suppressed cells after DMSO treatment. **(A,B)** Representative time-lapse SDCM images of the central volume of $HeLa_{rCDS}$ cells transfected with pCHIV and $pCHIV^{EGFP}$. Maximum intensity projections of four focal planes acquired with an axial spacing of 0.5 µm are shown. Cells were treated with 1 µM rCD1 at 5 hpt. Prior to imaging, cells were stained with 2G12FabSR for 1 h. At 22 hpt, cells were treated with 1 % DMSO for up to 90 min. Images of Env **(A)** and Gag **(B)** are displayed in the "Fire" LUT, which is shown in the upper right corner in the left panel in (A). Scale bars in overviews and enlargements represent 20 µm and 5 µm, respectively. Quantitative analysis is shown in Fig. 2G.

15

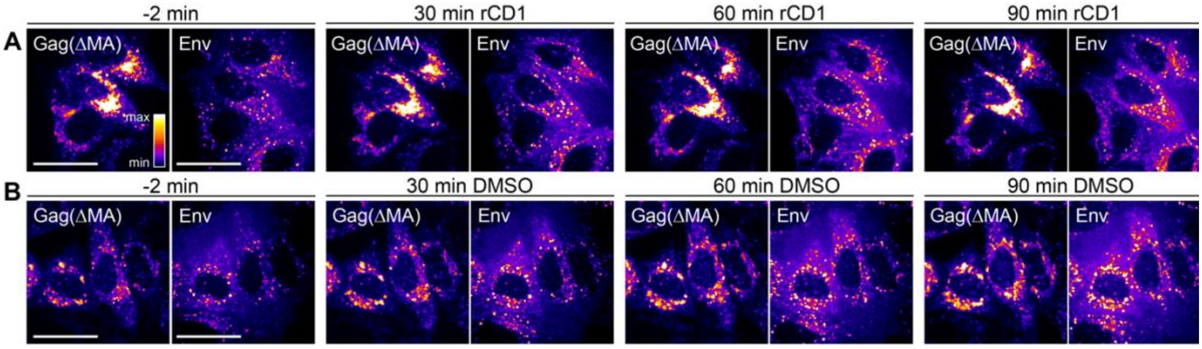

Fig S3 Loss of Env clusters depends on Gag MA. **(A,B)** Representative time-lapse SDCM images of the central volume of $HeLa_{rCDS}$ cells transfected with pCHIV(d8SR126) and $pCHIV^{EGFP}$(d8SR126). Maximum intensity projections of four focal planes acquired with an axial spacing of 0.5 µm are shown. Prior to imaging, cells were stained with 2G12FabSR for 30 min. At 22 hpt cells were treated with 1 µM rCD1 **(A)** or 1 % DMSO **(B)** for 90 min. Images of Env (large images) and Gag(dMA) (insets) are displayed in the "Fire" LUT, which is shown in the lower right corner in the left panel in (A). Scale bars represent 20 µm.

16

Movie S1 Env recruitment to nascent assembly sites**.** Representative SDCM high time resolution image series recorded at 10s/frame. Micrographs show the ventral PM of HeLarCDS cells transfected with pCHIV and pCHIVEGFP. The relevant cell is highlighted with outlines. At 4 hpt, the cells were treated with 1 µM rCD1. Cells were imaged at 22 hpt and 1 µM FK506 was added at t=0. Green, Gag-EGFP; magenta, Env(2G12FabSR). Scale bar represents 10 µm.

Movie S2 Env clusters dissociate with Gag after PM PI(4,5)P2 depletion. Representative SDCM image series of the central volume of HeLarCDS cells transfected with pCHIV and pCHIVEGFP. Gag (left) and Env (right) are shown. Maximum intensity projection of four focal planes acquired with an axial spacing of 0.5 µm. Prior to imaging, cells were labeled with 2G12FabSR for 30 min. At 22 hpt, cells were treated with 1 µM rCD1 for up to 120 min in imaging medium containing 2G12FabSR. Enlarged regions at the top are indicated as boxed regions (i/ii). Scale bar represents 20 µm.

Movie S3 Gag and Env clusters remain unaffected by DMSO treatment. Representative SDCM image series of the central volume of HeLarCDS cells transfected with pCHIV and pCHIVEGFP. Gag (left) and Env (right) are shown. Maximum intensity projection of four focal planes acquired with an axial spacing of 0.5 µm. Prior to imaging, cells were labeled with 2G12FabSR for 30 min. At 22 hpt, cells were treated with 1 % DMSO for up to 120 min in imaging medium containing 2G12FabSR. Enlarged regions at the top are indicated as boxed regions (i/ii). Scale bar represents 20 µm.
